# Generation and Analysis of the Expressed Sequence Tags from the Mycelium of *Ganoderma lucidum*

**DOI:** 10.1371/journal.pone.0061127

**Published:** 2013-05-02

**Authors:** Yen-Hua Huang, Hung-Yi Wu, Keh-Ming Wu, Tze-Tze Liu, Ruey-Fen Liou, Shih-Feng Tsai, Ming-Shi Shiao, Low-Tone Ho, Shean-Shong Tzean, Ueng-Cheng Yang

**Affiliations:** 1 Department of Biochemistry, Faculty of Medicine, School of Medicine, National Yang-Ming University, Taipei City, Taiwan, R.O.C.; 2 VYM Genome Research Center, National Yang-Ming University, Taipei City, Taiwan, R.O.C.; 3 Institute of Biomedical Informatics, College of Life Science, National Yang-Ming University, Taipei City, Taiwan, R.O.C.; 4 Center for Systems and Synthetic Biology, National Yang-Ming University, Taipei City, Taiwan, R.O.C.; 5 Department of Plant Pathology and Microbiology, National Taiwan University, Taipei City, Taiwan, R.O.C.; 6 Medical Research and Education Department, Taipei Veterans General Hospital, Taipei City, Taiwan, R.O.C.; American University in Cairo, Egypt

## Abstract

*Ganoderma lucidum* (*G. lucidum*) is a medicinal mushroom renowned in East Asia for its potential biological effects. To enable a systematic exploration of the genes associated with the various phenotypes of the fungus, the genome consortium of *G. lucidum* has carried out an expressed sequence tag (EST) sequencing project. Using a Sanger sequencing based approach, 47,285 ESTs were obtained from *in vitro* cultures of *G. lucidum* mycelium of various durations. These ESTs were further clustered and merged into 7,774 non-redundant expressed loci. The features of these expressed contigs were explored in terms of over-representation, alternative splicing, and natural antisense transcripts. Our results provide an invaluable information resource for exploring the *G. lucidum* transcriptome and its regulation. Many cases of the genes over-represented in fast-growing dikaryotic mycelium are closely related to growth, such as cell wall and bioactive compound synthesis. In addition, the EST-genome alignments containing putative cassette exons and retained introns were manually curated and then used to make inferences about the predominating splice-site recognition mechanism of *G. lucidum*. Moreover, a number of putative antisense transcripts have been pinpointed, from which we noticed that two cases are likely to reveal hitherto undiscovered biological pathways. To allow users to access the data and the initial analysis of the results of this project, a dedicated web site has been created at http://csb2.ym.edu.tw/est/.

## Introduction

*Ganoderma lucidum* (*G. lucidum*), which also has the trivial names Lingchi, Lingzhi, or Reishi, is a widely used medicinal mushroom in Asian countries. It has a long history of usage in the traditional Chinese medicine and holds a reputation of having enormous health and longevity benefits (for review see [1]). Over the past decades, substantial scientific evidence has accumulated to support the medicinal effects of *G. lucidum*. For example, polysaccharides extracts from *G. lucidum* have been shown to modulate mouse immunity and to help fight against cancers [2]. Certain triterpenoids and steroids from *G. lucidum* have also been found to have novel biological activities and pharmacological functions including antitumor cytotoxicity [1] and the inhibition of cholesterol synthesis [3]. Additionally, *G. lucidum* causes white-wood rot and is likely to play an important role in nutrient cycling due to its capacity to release an array of enzymes that are able to decompose lignin and cellulose from dead wood. It has been suggested that *G. lucidum* might produce as many as ∼400 different bioactive compounds [1]. This fungus has therefore been regarded as a treasury of novel compounds that may have both medicinal and industrial applications. *G. lucidum* is therefore an appealing species to biologists because of its aforementioned potential applications. *G. lucidum* is a multicellular fungus that also has remarkable features that are of evolutionary interest. To date, *G. lucidum* is one of the few fungi that can be induced to form fruiting bodies *in vitro*. Therefore, comparative studies of *G. lucidum* with other fungi may not only facilitate systematic studies of the molecular mechanisms implicated in synthesis of bioactive compounds, but also provide useful insights into the development of fruiting bodies by mushrooms. To this end, the Consortium of *Ganoderma lucidum* (CGL) has sequenced the genome of a *G. lucidum* strain (BCRC 37177) and the first genome assembly has just been finished (manuscript in preparation, contig sequence accession number [DDBJ: BACH01000001–BACH01003275]). Among the ∼13,000 predicted genes, about two-thirds of them can be functionally annotated. This genome project promises to provide a substantial contribution to the understanding of the systems biology of filamentous fungi.

The gene-structure assignment of the first *G. lucidum* genome assembly was largely dependent on *ab initio* gene finding algorithms. To provide experimental evidence to support gene-structure prediction, the CGL conducted a companion expressed-sequencing-tag (EST) sequencing project. Unlike previous fungal EST projects that did not focus on particular developmental stages [4], [5], this *G. lucidum* EST project concentrated on the initial 30-day mycelium stage. There are several reasons for this choice. First, the mycelium from *G. lucidum* at this point is in the early differentiation stage of initiating fruiting bodies. Transcripts expressed at this stage are more likely to be differentially regulated and alternatively spliced. Second, *G. lucidum* mycelium is known to produce bioactive substances with a degree of potency comparable to that of *G. lucidum* fruiting bodies [6]. Therefore, we believe that the ESTs collected during this stage will be able to provide valuable information about the initial development of *G. lucidum* as well as help to explore the molecular mechanisms involved in synthesizing bioactive compounds produced by *G. lucidum*. In this manuscript, we describe the experimental design and the analyses carried out by the CGL EST sequencing project, and then we discuss the biological significance of the findings; these relate to alternative splicing of transcripts, over-represented genes, pathway activation, and putative sense/antisense gene pairs.

## Materials and Methods

### mRNA extraction and cDNA library construction

Complementary DNA (cDNA) libraries of *G. lucidum* were constructed using the ZAP Express cDNA Synthesis Kit and ZAP Express cDNA Gigapack III Gold Cloning Kit (Stratagene Co. Ltd.). Messenger RNAs (mRNA) were primed in the first-strand synthesis with a hybrid oligo(dT)-linker primer that contained an *Xho* I restriction site. After the second-strand synthesis of cDNA, an *EcoR* I restriction site-containing adaptor was ligated to the blunt end. The *Xho* I and *EcoR* I restriction sites were used as the cloning sites to insert the cDNA into the pBK-CMV phagemid vector. The sequences of these two sites and the phagemid vector would be used later in “EST sequencing and post-processing” in order to remove vector sequences that are not a part of any *G. lucidum* genes.

Four cDNA libraries were prepared using RNAs extracted from mycelium of a *G. lucidum* dikaryotic strain, BCRC 36123, after cultured at 30°C for 5, 14, 18, and 30 days (for the detailed culture condition see [56]). The libraries were named 5D_36123, 14D_36123, 18D_36123, 30D_36123, respectively. In addition, a cDNA library, 18D_37180, was prepared from mycelium of *G. lucidum* BCRC 37180, a monokaryotic strain, after cultured for 18 days. The sources of the two *G. lucidum* strains and the growth conditions are available from the Bioresource Collection and Research Center, Taiwan (http://www.bcrc.firdi.org.tw/) [7]. When culturing *G. lucidum* fruiting bodies, the 1^st^-day to 18^th^-day stage is the period of time in which the *G. lucidum* mycelium grows and colonizes solid substrate medium, which is a prerequisite step for subsequent primordia formation [1]. Hence, certain ESTs generated within the mycelium at this stage ought to correspond to the differentiation-specific genes of *G. lucidum*. In addition to studying the differentiation of fungi, *G. lucidum* mycelium is also a suitable material for studying the synthesis of bioactive compounds. It is known that polysaccharides produced by *G. lucidum* mycelium cultivated for 30 days show relatively high biological activities against tumor cells [2]; *G. lucidum* mycelium also produces triterpenoids after being cultivated for 18 days (unpublished data by Dr. Ming-Shi Shiao). It is expected that the genes involved in the pathways responsible for the generation of these compounds would be expressed over this period. Therefore the cDNA libraries were non-normalized in an attempt to find over-represented *G. lucidum* genes that might be differentially expressed in the growth of mycelium. Differences in the relative abundance of ESTs among non-normalized libraries have been used to infer differentially expressed genes under certain conditions like human cancers [8], [9], [10], [11], [12], [13]. To reduce the chance of introducing bias due to the cloning of transcript of differential lengths or compositions across the different cDNA libraries, special attention was paid to manipulating them experimentally in a uniform manner.

### EST sequencing and post-processing (vector/adaptor strip, de-contamination and polyA/T removal)

The cDNA libraries prepared as described in the previous subsection were sequenced using the Dye terminator chemistry (Applied Biosystems BigDye Terminator v3.1) and capillary electrophoresis analysis (Applied Biosystems 3730xl DNA analyzer and GE Healthcare MegaBACE 1000). The workflow of generating the raw ESTs, their trimming, and then their filtering is as follows. The chromatograms generated in the ABI format were base-called into raw expressed sequence tags (ESTs) using Phred [14], [15], where low-quality bases were trimmed from the 5′ and 3′ ends of the raw ESTs using trace quality (Phred parameter trim_alt = 0.01). At this stage, the ends of many raw ESTs may still contain the adaptor and vector sequences used for cloning cDNAs. In addition to this, some raw ESTs contained polyA/T tracks at their ends, even though these tracks should have been removed during the construction of the cDNA libraries. Vectorstrip was used to trim the non-GL sequences such as the cloning adaptors and the vector remained in the raw ESTs [16]. The sequences of the *Xho* I and *EcoR* I restriction sites as well as the pBK-CMV phagemid vector (retrieved from the UniVec Database of NCBI) were given to Vectorstrip and the maximum allowed mismatch was set to 20%. Any remained PolyA/T track was trimmed along with its flanking sequence proximal to the end of the EST using a homemade Perl script. Next the trimmed ESTs were screened to detect contamination sequences by running a BLAST sequence similarity search against the *E. coli* genomes, phage genomes, mitochondrial genomes, and ribosomal RNAs (downloaded from NCBI GenBank). In each search, if the alignment of an EST to the non-GL sequences described above had an identity > = 0.90 and E-value< = 1e-10, this EST was regarded as contamination and was discarded. Any ESTs that remained after the trimming and decontamination were retained only if their length was longer than 50 bases.

### Aligning the ESTs to the reference genome and merging them into non-redundant sets

ESTs are a random sampling of a transcriptome where multiple ESTs could be derived from a same gene and thereby a set of ESTs could share a compatible splicing structure. The gene structure information dispersed in various EST-genome alignments cannot be directly compared with that of the genes predicted by *ab initio* methods (*i.e.* GeneMark-ES [17] was used in this project). To facilitate identification of alternative splice forms and the functional annotation of putative transcripts, redundant information on the splicing structures provided by ESTs must be merged. So far, two different approaches have been used by other groups to reconstruct non-redundant expressed contigs from ESTs [18]. EST self-clustering methods are the first choice when no reference genome for the species under investigation is available. On the other hand, genome-based methods, also called “splice alignment” algorithms [19], [20], [21], [22], are an alternative approach when there is a reference genome for the studied species. In the context of determining alternative splice forms, the latter approach is regarded as superior to the EST self-clustering methods. Splice alignment algorithms treat each EST independently and each EST is aligned separately to the reference genome. In other words, ESTs with different splicing structures are not merged in the first place, enabling the preservation of isoform information. Using these methods it is possible to distinguish alternative splice forms and the strandedness of transcripts can be inferred from consensus sequences at the splice junctions [18]. Genome-based approaches have been applied to finding transcript isoforms in a variety of eukaryote genome projects, including vertebrates, plants, and fungi [20], [23], [24].

With the availability of genome sequence of *G. lucidum*, we chose ClusterMerge to group ESTs into expressed contigs. ClusterMerge is a method that implements the genome-based approach and has used to build “ESTGenes” in Ensembl [18], [25]. In the preparation step of using the ClusterMerge method, the ESTs that remained after the previous trimming and filtering were initially aligned to the *G. lucidum* genome (BCRC 37177, accession numbers of the contigs: [DDBJ: BACH01000001–BACH01003275]) by Exonerate, which is a sequence alignment tool that can align ESTs to a reference genome with high accuracy allowing the exon-intron junctions to be determined [26]. Next, the EST-genome alignments were clustered and merged on the basis of sharing compatible splicing structures by the ClusterMerge method. The two EST-genome alignments to be merged must have at least one consecutive match of “splice-sites”, namely, the 5′ donor site and the 3′ acceptor site for an intronic region. Two splice-sites would be regarded as “matched” only if their coordinates are exactly the same. On the other hand, two overlapping EST-genome alignments were not eligible for merging if they did not have any shared splicing structures or if their splicing structures were inconsistent. For example, two EST-genome alignments would not be merged if one contains a skipped exon that does not exist in the other.

At this stage, the partial gene structures contained in the ESTs were merged into longer and non-redundant expressed contigs. These expressed contigs are referred to as ESTTranscripts in the following text, indicating that they were derived from clustered and merged ESTs but not sequenced from tentatively full-length cDNA clones. To facilitate the investigation of alternative splice forms and the gene structure evolution of *G. lucidum*, these ESTTranscripts were further grouped into ESTGenes. If a set of overlapping ESTTranscripts were on the same strand of one chromosomal locus, they would be grouped into one ESTGene even if these ESTTranscripts did not share the same splicing structure. The terminology used here does not exactly adhere to the convention used by Ensembl. In the Ensembl web site and the ClusterMerge method, ESTGenes had been used as a collective term to indicate the gene-structure information, including genes, transcripts, and even exons, derived from clustered and merged ESTs. For clarity in this manuscript, an “ESTGene” is used to describe a non-redundant group of overlapping ESTTranscripts.

ESTGenes were annotated using a two-pass procedure. First, if an ESTGene overlapped with a predicted gene, the similarity-based annotation of the latter one was directly used to name this ESTGene. All predicted genes had been pre-annotated using NCBI BLASTP [27] to search for their most similar genes in the UniProt database [28]. Second, if an ESTGene did not overlap with any predicted genes, they were then annotated using NCBI BLASTX to obtain the most similar genes in the UniProt database.

### Manual curation and filtering of the EST-genome alignments in order to identify reliable cassette exons and retained introns

Cassette exons (CE) and retained introns (RI) are the two dominant types of splice variations in eukaryotes [4]. Apart from predicting the potential consequences of these splice variations for protein sequences and functional domains, CEs and RIs have been used to infer the dominating mechanism used for splice-site recognition in a species [4]. Two different types of splice-site recognition mechanisms, intron definition (ID) and exon definition (ED), have been proposed in eukaryotes (for a review see [1]). ID refers to a splicing mechanism during which the spliceosomes recognize splice sites in pairs across the intron, whereas in ED, splice sites across the exon are recognized as a unit. To measure the relative importance of ED and ID in eukaryotes, McGuire *et al.* proposed using the fraction of cassette exons (CE fraction) [4]. The rationale can be described briefly as follows. It can be inferred that one mutation at a splice site can lead to different consequences depending on either ID or ED determining the recognition of this site. If the site is recognized by ID, then this mutation is more likely to cause the flanking intron to be retained (*i.e.* retained intron or RI) than to cause the flanking exon to be skipped. If the site is recognized by ED, then the reverse is favored. Skipped exons are also referred to as cassette exons in the literature. McGuire *et al.* defined the CE fraction as CE/(CE+RI) [4]. Therefore, a high CE fraction for a species suggests that splicing in this species is dominated by the ED mechanism, whereas a low CE fraction suggests a splicing mechanism in this species is dominated by the ID mechanism. Thereby, a similar measurement was performed in this study based on the *G. lucidum* EST-genome alignments.

One caveat for the direct usage of the automatically generated EST-genome alignments to find CEs and RIs is the accuracy. Due to the limitations of available splice-alignment algorithms, an EST containing sequencing errors, even if there are only a few of them, is likely to be misaligned relative to the genome. Putative CEs or RIs revealed by such an alignment could be false positives. To reduce this problem, the raw *G. lucidum* EST-genome alignments that appeared to contain CE or RI information were manually curated and then filtered. An EST-genome alignment was manually adjusted if an initially poorly aligned region, which contains multiple mismatches or indels, appears to be spurious. The aim of the adjustment was to decrease the number of indels in an EST-genome alignment without introducing additional mismatches. Next, the curated EST-genome alignments were filtered with the criteria already used in previous reports [4], [20]. An EST-genome alignment would not be used to infer CEs or RIs if it had non-canonical splice sites (*e.g.* GT:AG, CG:AG, or AT:AC was not present). In addition to this, continuous insertions or deletions outside of the introns must be shorter than 9 bases in length and each candidate CE or RI must be bounded by at least three exact matches in the EST-genome alignment.

### Assigning the functional categories and pathways of the predicted genes

To assign functional categories to the predicted genes, the protein sequences of other species that have been functionally categorized were downloaded from the Munich Information Center for Protein Sequences (MIPS) [29]. Next, the sequence similarity of each ESTGene to the MIPS categorized proteins was determined using NCBI BLASTP. The functional category of a gene was assigned using that of its most similar counterpart in the MIPS categorized proteins.

In addition, KAAS [30] was used to obtain the pathway assignment of each gene. The putative protein sequences of the predicted genes were submitted to KAAS, which then returned the mapping of each gene to its corresponding entity in the KEGG pathways [31].

### Comparing the sequences of BCRC 37177 with those of another two strains of G. lucidum

To explore the differences among the three sequenced strains of *G. lucidum* thus far, we compared their genomes and the proteins coded in them. The genomic sequences of another two *G. lucidum* strains, Xiangnong No. 1 [GenBank: AHGX01000001.1–AHGX01001708.1] [32] and G.260125-1 [GenBank: AGAX01000001.1–AGAX01000194.1] [33] were retrieved from GenBank. The protein sequences predicted in the genome of G.260125-1 were downloaded from http://www.herbalgenomics.org/galudb/analyzer/download. To compare the genomic sequences of the strain (BCRC 37177) used in this study with those of Xiangnong No. 1 and G.260125-1, MUMmer 3 was used [34]. With respect to comparing the coding regions of different strains, we did not directly compare the protein sequences predicted in different strains by using BLAST-like tools. We are aware that the genes and their structures predicted by distinct *ab initio* gene-finding algorithms often differ to a certain extent (for review see [35]). This means that the differences that can be revealed by directly comparing predicted proteins of one strain with those of another strain may occasionally reflect the varied performances of different algorithms. Therefore, we chose Exonerate to align the protein sequences predicted in one strain, either BCRC 37177 or G.260125-1, to the genomic sequences of the other strains. The protein2genome mode of Exonerate was used in order to allow introns and frameshifts in the protein-to-genome alignments [26]. The comparison result was also used to build a lookup table for the best inter-strain similar protein coding genes implicated in certain pathways of biomedical importance, such as the synthesis of *N*-glycans and triterpenoids. If the best similar counterparts of a gene do not have a high similarity at the protein sequence level (> = 90%), NCBI TBLASTN was used to confirm that no other coding regions with higher similarities have been missed due to the performance limitation of Exonerate.

## Results

### The CGL ESTs and contigs of ESTs

Using PCR to amplify the inserts of randomly selected clones and then performing DNA gel electrophoresis, the insert sizes of the cDNA libraries were estimated to be 0.5 kb∼3.2 kb for 5D_36123, 14D_36123, 18D_36123, and 30D_36123, and 0.6 kb∼3.0 kb for 18D_37180. The sequencing results generated a total of 53,323 sequencing chromatograms in the ABI format and all of them were base-called into raw expressed sequence tags (ESTs). After trimming low-quality and adaptor-and-vector sequences, removing polyA/T tracks, and then decontaminating the sequences, a set of 47,285 ESTs remained (∼89% of 53,323 chromatograms) (see Table 1 for the statistics of these libraries). The lengths of remaining ESTs range from 50 to 949 bases, with the median at 729 bases, the first quantile at 593 bases, and the third quantile at 793 bases (for the length distribution of the ESTs see File S1). These ESTs have been submitted to the DNA DataBank of Japan (DDBJ), which is part of the International Nucleotide Sequence Database Collaboration. In order to further analyze these ESTs [accession numbers: DDBJ: HO710205–HO757489], they were aligned to the contigs of the reference genome (BCRC 37177) [accession numbers: DDBJ: BACH01000001–BACH01003275] as described in the Materials and Methods. To allow other groups to access the results presented in this manuscript, both the genomic contigs and all the ESTs will be released to the public once the manuscript is accepted, and thus any users can retrieve these sequences from DDBJ/EMBL/GenBank.

**Table 1:** The number of ESTs obtained from the various mycelia that had been cultured for a different number of days.

EST library	# filtered EST	# CGL ESTs (GL-mapped)	# Spliced ESTs
5D_36123 (dikaryon)	1,001	964	828
14D_36123 (dikaryon)	6,848	6,701	5,820
18D_36123 (dikaryon)	21,547	21,078	17,455
18D_37180 (monokaryon)	11,059	10,782	8,944
30D_36123 (dikaryon)	6,830	6,628	5,348
Total	47,285	46,153	38,395

The result of the EST-genome alignments suggest that the trimming and de-contamination process had effectively removed most non-*G. lucidum* sequences. Nearly all of the ESTs (47,283 out of 47,285) could be mapped onto the *G. lucidum* genome by Exonerate [26], where about 98% (46,153 out of 47,283, see Table 1) of the EST-genome alignments had both the identity and coverage of no less than 90%. This result suggests that most of the sequences of these ESTs are of high quality and contain only a few genome-unalignable regions that might be caused by sequencing errors, cloning adaptors, or cloning vectors. These 46,153 ESTs are referred to as the CGL ESTs (GL-mapped ESTs in Table 1). About 83% of the EST-genome alignments (38,395, “Spliced ESTs” in Table 1) contained gaps that are likely to be introns in multi-exon genes. Most (∼99.9%, 38,347/38,395) of the putative introns were shorter than 2 kb, with a median length of ∼280 bases and a shortest length of 24 bases. These splice-junction-spanning ESTs provided invaluable information about the gene structures of *G. lucidum*, and their alignments with the reference genome were clustered and merged to generate 8,836 non-redundant expressed contigs, which are referred to as ESTTranscripts in the following text. These ESTTranscripts were further grouped into 7,774 non-redundant ESTGenes, which were annotated using a two-pass procedure as described in Materials and Methods.

In the remaining part of the results section, we first compare the structures of the predicted genes with those inferred from the CGL ESTs. Then we analyze the non-redundant ESTTranscripts and ESTGenes in order to explore the alternative splice forms and the associated features found in these genes together with a survey of the putative sense-and-antisense gene pairs. Finally, we describe the *G. lucidum* EST-relevant materials and resources that can be accessed at the Lingzhi EST project web site (http://csb2.ym.edu.tw/est/) and the Ensembl-based companion site.

### A summary of the findings about the CGL ESTTranscripts and ESTGenes

To outline the findings in this study, we summarize important statistics about the ESTTranscripts and ESTGenes as follows:

Alternative splice forms:
  262 ESTGenes contain alternative splice forms, among which 16 genes contain skipped exon(s) and 191 genes contain retained intron(s).
ESTGenes that were unpredicted by GeneMark-ES:
  About six hundred ESTGenes were missed in the initial gene prediction made by GeneMark-ES. 484 GeneMark-ES-missed genes contain at least one canonical GT-AG splicing junction and 116 of them could be functionally annotated.
Anti-sense transcripts
  There are 101 pairs of anti-sense transcripts, where 68 pairs are convergent and 7 pairs are divergent. In each of the other 28 pairs it was found that one forward transcript fully covers the other reverse and complement transcript. Besides, 51 pairs consist of both protein-coding genes and 35 pairs of them belonged to pure 3′-UTR type.
Over-represented GeneMark-ES-predicted genes
  There are 108 over-represented genes that were supported by at least 50 ESTs, where 44 predicted genes were supported by significantly more age-specific ESTs.

### Gene-structure comparison between ESTs and GeneMark-ES prediction

The gene structures revealed by the CGL ESTs showed a moderate level of difference from those of the GeneMark-ES prediction. At the exon level, about 86% (30,784) of the CGL EST exons (35,972) overlap with the GenMark-ES-predicted exons, while about 50% (15,158/30,784) of the CGL exons matched exactly the predicted exons. At the transcript level, about 16% (1,274/7,774) of the ESTGenes consisted of the same exon-intron structures as predicted by GeneMark-ES at their respective genomic loci. These 16% of the ESTGenes were constructed from more than one-third (16,981/46,153) of all CGL ESTs. This result agrees with the hypothesis that highly expressed genes are cloned and sequenced more frequently, and that their gene structures are more likely to be fully reconstructed from partially expressed sequences (ESTs).

In addition, the gene-level consistency between ESTGenes and predicted genes was assessed. Our result indicates that GeneMark-ES showed high sensitivity when pinpointing the coding loci in the *G. lucidum* genome because about 91% (7,112) of the ESTGenes (7,774) overlap with 6,037 of the GeneMark-ES-predicted genes. However, it is impossible to overlook the fact that this mapping does not represent a strict one-to-one relationship. Multiple ESTGenes could be mapped onto non-overlapping regions of a single predicted gene. The possible reasons for this discrepancy include: 1) some predicted genes might encompass exons of neighboring genes and 2) the ESTs cloned in this project were insufficient to span the full length of corresponding transcripts/genes.

In genome sequencing projects, ESTs are a valuable resource since they may contain initially unpredicted genes as well as alternative splice forms that are generally missed by *ab initio* gene-finding algorithms. These are the topics that are explored in the following two subsections.

### ESTGenes that are unpredicted by GeneMark-ES

The list of ESTGenes contains an additional ∼600 genes that were not found by GeneMark-ES. Among these genes, 484 of them had at least one canonical GT-AG splicing junction (for the gene list see File S2), thus are unlikely to be just a consequence of contamination or other incidental errors during the EST cloning. Although the number of such gene-finder-missed genes is small, their contribution to extending our knowledge about the *G. lucidum* transcriptome might be significant. Some of these genes may be species-specific or noncoding RNA genes. Less than a quarter (116/484) of these genes were found to have similar genes in other species. This ratio is significantly lower than that (10,934/13,681) of the GeneMark-ES-predicted genes. In addition, some of these genes may be only transiently expressed at a particular stage during development. In this context, about 62% (300/484) of these genes were single-EST supported and thus their expression might be regulated developmentally and/or involve a novel regulatory role.

### Alternative splice forms and the major mechanism of splice-site recognition in G. lucidum

From the 7,774 ESTGenes we have collected, 262 such genes may have alternative splice forms, including cases showing 5′ and/or 3′ alternative splice junctions, cassette exons, and retained introns. To compare this result with that of another fungal species, we used a variant-EST index (VE index) where the number of variants is divided by the number of ESTs derived for each species, as an indicator of the relative abundance of alternative splicing information. The VE index for the CGL ESTs was ∼0.006 (262 splice variants/46,153 ESTs), a moderate value when compared with other EST sequencing projects for fungi (*e.g.* ∼0.018 for *C. neoformans* (1,091 variants/59,041 ESTs), ∼0.003 for *M. grisea* (151 variants/53,102 ESTs) [4]).

These alternative splice forms not only increase our understanding of the transcriptome of *G. lucidum*, but also allow us to infer the major mechanism involved in splice-site recognition in this species. For this purpose, we use the fraction of cassette exons (CE fraction, defined as CE/(CE+RI), where CE refers to cassette exon and RI refers to retained intron [4]). In the *G. lucidum* EST-genome alignments, it was found that 16 genes seem to contain at least one CE; this involves 26 exons. However, 191 genes seemed to contain at least one RI; this involves 246 exons. Among the CEs, 11 of them were found in a transcript isoform as a stretch of consecutive exons that were skipped simultaneously (for their EST-genome alignment see YMGLESTG51605.aln in File S7).

Thus, the CE fraction of *G. lucidum* is about 0.10 (CE = 26, RI = 246). This fraction is much lower than those of animals (0.28∼0.95). Based on this measure, the splice-site recognition in *G. lucidum* should be dominated by intron definition (ID), a feature that is similar to the situation in other fungi [4]. *G. lucidum*'s CE fraction is the third highest in fungi (0∼0.21, median = 0.02). The count of cassette exons reported in this study is the highest so far reported for other fungi. The cassette exons are unlikely to be false positives caused by spurious EST-genome alignments resulting from cloning or sequencing errors. All these cassette exons and retained introns as well as their flanking exons are supported by high-quality EST-genome alignments with high sequence identities. In addition, all the splicing junctions in the EST-genome alignments containing CEs or RIs must be canonical. Their EST-genome alignments are provided in File S7 and File S8, respectively.

### Over-represented genes in different libraries

Since the *G. lucidum* ESTs were derived from non-normalized libraries, the counts of ESTs sequenced for a gene may, to a certain extent, reflect the abundance of the transcripts of this gene under a particular condition. Hence we took the counts of ESTs as a semi-quantitative measurement and used this to search for over-represented genes in different libraries. Genes significantly over-represented in certain libraries are likely to correspond to differentially expressed genes, even though we cannot rule out the possibility that some cases could be false positives due to the insufficient depth of coverage in EST sequencing.

As an initial assessment of the overall distribution of ESTs over different genes, the counts of supporting ESTs *versus* GeneMark-ES predicted genes are summarized in Table 2. There are 108 predicted genes in which each is supported by at least 50 ESTs (for the genes see File S3). Noticeably, these ESTs amount to around one-fifth (9,777/46,798) of all CGL ESTs. This result is consistent with the fact that the *G. lucidum* cDNA libraries prepared in this study were non-normalized.

**Table 2:** Summary of the predicted genes supported by ESTs.

# Supporting ESTs	# Predicted genes
0	6,912
1–49	7,033
50–99	85
100–149	13
150–199	5
200–249	2
250–299	0
300–349	0
350–399	2
579	1

All the CGL ESTs were then used to pinpoint candidate genes that might be over-represented at particular stages during *G. lucidum* development. We found that 44 predicted genes were supported by significantly more ESTs cloned from mycelium at specific times during culture (Chi-square test in R, simulated *p*-value<1e-5) (for the genes see File S4). These genes are likely to be implicated in the development and/or differentiation of *G. lucidum* mycelium and further investigation is needed in order to elucidate their roles. This objective is likely to be fairly difficult at the present time because about 40% (18/44) of these have no known function. Additionally, we found 39 genes that are over-represented either in the monokaryotic strain (BCRC 37180) or in the dikaryotic strain (BCRC 36123) (using the binomial test, two-sided *p*-value<1e-5) (for the genes see File S5). It should be noted that the genes found to be over-represented in the libraries do not necessarily correspond to differentially expressed genes. Gene representation in one library but not another could just reflect the situation that the depth of the sequencing is low that not all expressed transcripts could be sampled. However, we do observe some interesting cases that could be correlated to the growth of mycelium as well as the synthesis of bioactive compounds. These cases are further discussed later in “Discussion”.

### Antisense transcripts

*Cis*-natural antisense transcripts (*cis*-NATs) have been found in most eukaryotes and they have been shown to play regulatory roles in gene expression (for reivew see [36]). To explore the incidence and regulation of *cis*-NATs in the *G. lucidum* genome, we looked for pairs of overlapping but reversely oriented transcripts among the *G. lucidum* ESTGenes. To avoid possible biases caused by incorrect strand assignment, which was automatically made by Exonerate, we excluded genes that did not contain any canonical GT-AG splicing junctions. In this way 101 high-confidence candidates for sense and *cis*-NAT pairs (SCPs) were collected (for the SCPs see File S6). This number is similar to the intron-containing SCPs (101) found in another fungus, *Aspergillus flavus* (*A. flavus*) [36]. About 67% (68/101) of the *G. lucidum* SCPs were convergent, with the sense and antisense members in the pair arranged in a tail-to-tail manner. The abundance of convergent SCPs is consistent with the current view that this type of SCPs prevails across kingdoms including fungi [36]. Furthermore, this ratio is higher than that estimated for other fungi such as *A. flavus* (46%) [36].

**Figure 1:**
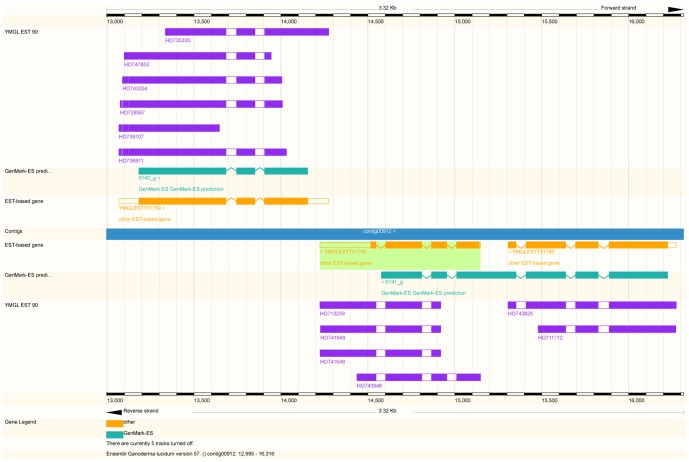
A tail-to-tail arrangement of sense and *cis*-NAT gene pairs, *CCHL* (YMGL ESTG/ESTT 51764) and *TIM44* (YMGL ESTG/ESTT 51765). This figure presents an Ensembl-based visualization of the ESTs and ESTGenes/ESTTranscripts, YMGL ESTG/ESTT 51764 and YMGL ESTG/ESTT 51765, on top of *G. lucidum* genomic contig, contig00912. The former is annotated as cytochrome c heme lyase (*CCHL*) and the latter is annotated as mitochondrial import inner membrane translocase, subunit Tim44 (*TIM44*). The EST transcripts were built from overlapping ESTs-genome using a “cluster and merge” algorithm (see the Materials and Methods for details) and only the ESTs sharing identical splicing junctions were merged into an ESTTranscript. The orientation of each transcript was inferred by fitting splicing junctions to the consensus GT-AG pattern. YMGL ESTG/ESTT 51764 and YMGL ESTG/ESTT 51765 are on opposite strands and their 3′ regions corresponding to putative untranslated regions that overlap with each other (as indicated by the non-shaded area in each transcript). These two transcripts show a tail-to-tail arrangement between the sense and *cis*-natural antisense transcripts.

In the context of protein-coding capability, convergent SCPs in the *G. lucidum* genome showed several interesting features. First, protein-coding genes, but not noncoding RNAs, appeared to dominate among the *cis*-NATs in the convergent SCP pool. In about 75% (51/68) of the convergent SCPs, both the sense and antisense members of each pair are significantly similar to protein-coding genes in other species (using NCBI BLASTX, E-value<1e-10). This ratio is higher than in previous reports describing SCPs in other fungi [36]. However, we are unable to rule out the possibility that *G. lucidum* mycelium may express a higher ratio of noncoding antisense transcripts when grown under particular conditions. In addition, the complementary regions of many convergent SCPs are restricted mainly to their respective 3′-untranslated regions (3′-UTRs). As many as 70% (35/51) of the convergent and protein-coding SCPs belonged to the pure 3′-UTR type, where the coding region in each transcript in the SCP is not involved (for an example see Figure 1). This result suggests that transcription regulation of the genes by these SCPs is likely to rely on mechanisms associated exclusively with the 3′-UTRs. The implied regulatory mechanisms thus may include translation repression, alternative polyadenylation, and mRNA degradation [37].

The tail-to-tail arrangement of a sense and antisense pair has been proposed as a mechanism for keeping functionally associated genes together as a co-regulated unit [36]. Below, we demonstrate two examples of convergent, pure 3′-UTR-type SCPs that help to suggest how genes are co-regulated in biological pathways in order to achieve a desired biological function.

The first example consists of YMGLESTG51764 (transcript ID: YMGLESTT51764) and YMGLESTG51765 (transcript ID: YMGLESTT51765), which correspond to the genes coding for cytochrome c heme lyase (*CCHL*) and mitochondrial import inner membrane translocase subunit 44 (*TIM44*), respectively (see the Ensembl ContigView as in Figure 1 for their gene structures, EST evidence, and complementary regions). The color-shaded areas on the “GeneMark-ES prediction” and “EST-based gene tracks” in Figure 1 indicate putative protein-coding regions and it can be seen that the complementary regions are exclusively within the 3′ UTRs. *CCHL* is essential for the import of apocytochrome c into the intermembrane space of the mitochondria in fungi [38], [39], while *TIM44* is important for the assembly of the translocation channels in the inner membrane of mitochondria [40]. An interesting conjecture is that this SCP may represent a novel mechanism whereby *G. lucidum* is able to indirectly orchestrate the synthesis of cytochrome c, and the import of other required proteins into the matrix of mitochondria. Perhaps this SCP ensures that the electron transport chain and oxidative phosphorylation are effectively coupled with each other across the mitochondrial inner membrane.

Another example of convergent SCPs consists of YMGLESTG47675 (transcript ID: YMGLESTT47675) and YMGLESTG47676 (transcript ID: YMGLESTT47676), which correspond to the genes coding for a pre-mRNA-splicing factor (*SPF27*) and RNA exonuclease 4 (*Rex4*), respectively. ESTs cloned for these two genes showed a co-expressed pattern on the 14^th^, 18^th^, and 30^th^ days of *G. lucidum* mycelium culture (see also the “SCP list” as mentioned above for the numbers of ESTs). This finding suggests that *Rex4* may play a role in pre-mRNA splicing. An appealing argument is that *Rex4* might be responsible for the degradation of spliced introns, a step that has been generally expected but never explicitly reported elsewhere in other species (for review see [41]).

## Discussion

The transcriptome of *G. lucidum* has not been extensively sequenced until this study. Only about 1000 messenger RNA sequences of *G. lucidum* genes had been deposited in GenBank before the completion of this study. Furthermore, the strain of the *G. lucidum* used to sequence those ESTs has not been explicitly mentioned nor is its reference genome available to the public [5]. It is therefore difficult to infer splicing junctions or other interesting features such as splice-site recognition mechanisms from those ESTs. Compared to previous studies, this *G. lucidum* EST sequencing project carried out a deeper sampling of the transcriptome in order to facilitate further annotation and future curation of the gene structures predicted from the *G. lucidum* genomic sequence. Using an improved Sanger sequencing method, this *G. lucidum* EST sequencing project adds ∼46,000 ESTs, with an average length of about 720 bases, to the public domain. When aligned to the reference genome, a high proportion of these ESTs have been found to span splicing junctions. The EST-genome alignments were further clustered and merged into about 8,800 expressed contigs (*i.e.* ESTTranscripts). These contigs provide information about the expression and gene structures of about 6,000 predicted genes and form a valuable resource for studying *G. lucidum*.

This resource will facilitate investigation of the biological pathways implicated in the synthesis of bioactive compounds. For example, the counts of ESTs provide a way for us to find candidate genes that could be differentially expressed during the fast growing stage of mycelium. To demonstrate how counts of ESTs might correlate with the phenotypes of the cultured *G. lucidum* mycelium, the “amino sugar and nucleotide sugar metabolism” pathway is used here as an example (for the diagram see http://csb2.ym.edu.tw/cgi-bin/est/show_pathway.cgi?id=00520& org=gl). This pathway is likely to be an active pathway in mycelium since about 81% (43/53) of the unique entities in this pathway are supported by the CGL ESTs. Two proteins, glutamine-fructose-6-phosphate amidotransferase (*GFA1*, EC number 2.6.1.16) and UDP-N-acetylglucosamine pyrophosphorylase (*UAP1*, EC number 2.7.7.23), are supported by 83 and 58 ESTs, respectively, from the 18^th^-day dikaryotic mycelium. These counts significantly deviate from the expected values. This result implies that these two proteins are likely to play important roles in the growth and development of dikaryotic mycelium. By contrast, *UAP1* is supported by only 3 ESTs derived from the 18^th^-day monokaryotic mycelium, which suggests a relatively low expression level in the monokaryotic mycelium.

It is possible to argue that this finding agrees with the phenotypes that are observed in the *G. lucidum* mycelium in two ways. First, it seems to reflect the fast growth of dikaryotic mycelium at 18 days. *GFA1* is the first and rate-limiting enzyme in the synthesis of chitin, which is one of the major structural components of fungal cell walls. In addition, in the same pathway, *UAP1* catalyzes the 4^th^ reaction, which generates the activated form of GlcNAc, UDP-GlcNAc (for review see [42]). Over-representation of these two genes on the 18^th^-day of dikaryotic mycelium growth is consistent with that there is greatly increased cell-wall synthesis at this time, a phenotype expected for fast-growing mycelium. Second, this finding might also reflect the growth of the 18^th^-day old monokaryotic mycelium. The relatively low abundance of ESTs found for UAP1 in the 18^th^-day monokaryon mycelium suggests that the transcription of UAP1 is not particularly activated, which is consistent with the fact that monokaryon strains generally grow at a much slower rate than dikaryon strains.

In addition to finding over-represented genes that are likely to be implicated in the development and differentiation of *G. lucidum* mycelium, we noticed that the EST information supports that certain metabolic pathways are likely to be activated at a particular stage during the fast growing of mycelium. For instance, a series of *G. lucidum* enzymes involved in the biosynthesis pathway of triterpenoid backbones, namely from 3-hydroxy-3-methylglutaryl-CoA synthase to farnesyl pyrophosphate synthase (see Table 3 for the gene list of these enzymes and the counts of ESTs supporting their expression), are mainly supported by ESTs derived from the 18^th^-day dikaryotic mycelium. In contrast, a very small fraction of ESTs (12 out of 224 ESTs) mapped to this pathway are derived from 5^th^- and 14^th^-day dikaryotic mycelium. The results suggest that the biosynthesis of the triterpenoid backbone is fully functioning after the mycelium has been cultivated for 14 days. The expression of squalene synthase, which converts farnesyl pyrophosphate into squalene, was also detected in *G. lucidum* after the 14^th^ day (Table 3, YMGLESTG51888). Based on this, we suggest that the period from the 14^th^ day to the 18^th^ day corresponds to a stage when the triterpenoid synthesis is activated. This speculation is consistent with previous findings from studies that investigated triterpenoids generated by *G. lucidum* mycelium, whereby it was found that the 18^th^ day was the earliest time point that triterpenoids could be detected in cultivated mycelium, although higher levels of triterpenoids are usually found in mycelium cultivated for a longer period of time, such as 30 days (unpublished data by Dr. Ming-Shi Shiao).

**Table 3:** *G. lucidum* genes and their ESTs involved in the biosynthesis of triterpenoids.

Gene_ID	Description	EC number	E_value	5^th^ day	14^th^ day	18^th^ day	*18^th^ day	30^th^ day
YMGLESTG49648	acetyl-CoA acetyltransferase	2.3.1.9	1.00E-112	0	5	33	3	12
YMGLESTG52067	3-hydroxy-3-methylglutaryl-CoA synthase	2.3.3.10	1.00E-121	0	2	68	9	12
YMGLESTG53675	3-hydroxy-3-methylglutaryl-coenzyme A reductase	1.1.1.34	0	0	0	16	1	4
YMGLESTG52428	beta-cystathionase (containing a mevalonate kinase domain)	4.4.1.8 (2.7.1.36)	1.00E-102	0	0	1	1	1
YMGLESTG50562	phosphomevalonate kinase	2.7.4.2	6.00E-58	0	0	2	0	0
YMGLESTG50973	mevalonate pyrophosphate decarboxylase	4.1.1.33	1.00E-112	0	1	5	1	1
YMGLESTG47454	isopentenyl-diphosphate isomerase	5.3.3.2	5.00E-91	0	4	3	0	6
YMGLESTG55240	farnesyl pyrophosphate synthase	2.5.1.1	1.00E-111	0	0	8	0	2
YMGLESTG52116	farnesyl pyrophosphate synthase	2.5.1.1	1.00E-117	0	0	11	4	8
YMGLESTG51888	squalene synthase	2.5.1.21	1.00E-124	0	2	3	5	4

EC number: Enzyme commission number.

The 5^th^-day, 14^th^-day, 18^th^-day, and 30^th^-day columns list the numbers of ESTs derived from the *G. lucidum* mycelia cultivated for corresponding days.

* : Derived from the monokaryotic strain, BCRC 37180.

E_value: The NCBI BLASTP E-value reported for the most similar gene in the non-redundant (NR) database for each putative protein.

In addition to correlating with the activation of biosynthesis of triterpenoid backbone in the mycelium cultivated for 14 days, these ESTs provide useful insights into the biosynthesis pathways of polysaccharides in fungi. *G. lucidum* is predicted to have a series of enzymes implicated in the precursor biosynthesis of *N*-glycans, which are the main constituents of glycoproteins in eukaryotes including fungi. The ESTs support the hypothesis that the mycelium of *G. lucidum* is able to express a complete set of enzymes required for synthesizing the core structure of *N*-glycans, namely tetradecasaccharide (for the genes and supporting ESTs see File S9) (for review see [43]). The core structure then is likely to be further modified to form more complex structures. For example, a number of fucosyltransferases have been experimentally characterized that are implicated in the extension of the *N*-glycan core structures in animals, plants, and even bacteria (for review see [44]). Surprisingly, we notice that none of the *G. lucidum* predicted genes and ESTGenes could be unequivocally implicated in the extension of the core glycan structures. The lack of such enzymes in the genome annotation is also observed in other fungi such as *S. cerevisiae*. This finding is inconsistent with the fact that *G. lucidum*, as well as other fungi, is able to synthesize glycoproteins containing monosaccharide residues in addition to glucose, mannose, and GlcNAc [45], [46]. Our results, together with previous findings and reports, suggest that a part of the biosynthesis pathway of *N*-glycans in fungi remains unexplored. Since a fucose-containing glycoprotein fraction of *G. lucidum* has been shown to stimulate spleen cell proliferation and cytokine expression *in vitro* [45], it would be worth characterizing the specificities of these glycosyltransferases in relation to a variety of monosaccharides.

In addition to polysaccharides and triterpenoids, fungi like *G. lucidum* can synthesize various proteins with interesting properties. The ESTs generated in this study provide valuable information about their expression, which could give us clues to the hitherto undiscovered roles of these genes. To demonstrate this point, the case of hydrophobins, which are a family of proteins unique to the fungal kingdom, can be explored. These proteins are important for fungi and are involved in interacting with the environments. Different hydrophobins show a range of hydrophobicity that stretches from moderate to high levels and these proteins may be involved in surface modification and emulsification [47]. Hydrophobins have been shown to self-assemble into an amphiphatic membrane that covers the outer-wall surface of hyphae at hydrophilic-hydrophobic or air-medium interfaces (for review see [48]). In *G. lucidum*, there are six distinct genes that belong to the *SC3* gene family. Each of genes was exclusively supported by the presence of certain ESTs (for the hydrophobin genes see File S10, and for the protein sequence alignment see File S11). Interestingly, some *SC3* genes appear to be over-represented in certain libraries, which suggests that they are likely to be differentially expressed under different conditions. For example, over fifty ESTs were found to support the expression of one *SC3* gene (see the ESTGene YMGLESTG53555 in File S10) and, significantly, most of the ESTs for this gene were identified from the 18^th^-day monokaryotic mycelium. By way of contrast, the ESTs of another *SC3* gene (see the ESTGene YMGLESTG53558 in File S10) were only found in dikaryotic mycelium from the 14^th^- and 18^th^-day. These findings suggest that the proteins coded by these two *SC3* genes may play distinctly different biological roles in monokaryons and dikaryons. Previous studies about SC3 proteins have been mainly focused on the fungus, *Schizophyllum commune*, in which there is only one *SC3* gene. SC3 in *S. commune* has been implicated in the formation of aerial hyphae both in monokaryons and in dikaryons [49]. However, the multiple *SC3* genes in *G. lucidum* and their changing representation patterns in the differently timed libraries creates a scenario that is quite different to that proposed for *S. commune*. It is possible in *G. lucidum* that the differences in sequences across the various SC3 proteins might result in proteins with distinct physicochemical characteristics, which is consistent with the viewpoint proposed for other hydrophobins in previous studies [50]. It would be interesting to explore how the different *SC3* genes are regulated and what significance their expression and function has during the growth or the mating of *G. lucidum* mycelium.

### Weaknesses of this study

Due to the high cost of the present approach, generating more ESTs for the purpose of further exploring expression profiles is not really practical. So far, there are only ∼1000 ESTs that were derived from the 5^th^-day mycelium, which is only about 5 percent of the ESTs derived from the 18^th^-day mycelium. In addition, the ESTs derived from the 14^th^- and 30^th^-day mycelium are also much fewer than the ESTs derived from the 18^th^-day mycelium. This causes two problems. First, the number of ESTs is so small that it is quite possible that only a small portion of all expressed sequences have been covered. Second, the statistical power when pinpointing differentially expressed genes is limited since there is a huge difference in the EST numbers between the samples. This means that, in addition to the cases found in this project, there are likely to be a significant number of differentially expressed genes that have as yet not been discovered. Therefore, to investigate the regulation and differentiation of *G. lucidum* more comprehensively in the future, we plan to perform RNA-seq, which is a genome-wide approach for identifying expressed sequences using the next-generation sequencing technologies. This will allow us to better explore the transcriptomes of *G. lucidum* at different developmental stages.

### GL's “cassette exon” fraction is influenced by how consecutively skipped exons are counted

The “cassette exon” (CE) fraction of *G. lucidum* is estimated to be 0.10, a value that is much lower than the estimates from animals [4]. This result is consistent with the previous finding that intron definition (ID) predominates as the means of recognition of splice sites in fungi. Interestingly, the initial estimate of *G. lucidum*'s CE fraction is higher than those of many other fungi (0∼0.21, median = 0.02) except for *Ustilago maydis* and *Neurospora crassa* [4]. The latter two species can be regarded as special cases since they have much fewer CEs and RIs than *G. lucidum*. *G. lucidum* has twice as many CEs as the sum of the CEs found in both the above fungal species (8 CEs and 31 RIs for *U. maydis*; 2 CEs and 8 RIs for *N. crassa*). We tried to use the McGuire *et al.*'s linear model to see how much the *G. lucidum*'s CE fraction exceeds expectation [4]. The linear model can be expressed as y = 0.84x+0.00, where y refers to the CE fraction and x refers to percentage of introns >200 bases. The implicit rationale of this model is that, if the distance between the two ends of an intron is greater than a certain threshold, they are less likely to be recognized as a pair by the spliceosome, implying that the two ends are independently recognized by exon definition (ED). Briefly, this model implies that in species with more long introns (>200 bases), the ED approach is more likely to play a major role in splice-site recognition. As 5% of *G. lucidum*'s introns in the predicted genes are longer than 200 bases, the expected value for its CE fraction is estimated to be 0.04. It is clear that the observed CE fraction deviates from the prediction and does so by more than 2 standard errors.

We notice that the estimation of the CE fraction can be greatly influenced by the way that consecutively skipped exons (CSEs) are counted to give the final CE count. This is an issue that was not explicitly discussed by McGuire *et al.* when they proposed the system for estimating the CE fraction and it is unclear how CSEs were used in their paper [4]. Hence, for the initial estimation of *G. lucidum*'s CE fraction in this study, each exon in a stretch of CSEs was regarded as a single-skipped exon. However, it can be envisioned that this approach would easily inflate the CE fraction because of the presence of a long stretch of CSEs in a transcript isoform. For example, there is a stretch of 11 CSEs in the ESTGene YMGLESTG51605 (for the EST-genome alignment see File S7). In a species like *G. lucidum* where there are only tens of single-skipped exons (SSEs), adding up the raw count of these CSEs can result in a significant increase in final CE count. Obviously, there is a need to reconsidered how CSEs should be used when calculating a CE fractions.

When using CE fractions in the manner of McGuire *et al.*, it is assumed that the pair of splicing sites across each CE or RI is recognized as a unit by a spliceosome [4]. Two distinct types of “paired-sites” recognition have been experimentally demonstrated, one for single-exon skipping [51], [52] and another for single-intron retention [53], [54]. Therefore, each event of single-exon skipping and intron retention is equally informative when inferring the relative importance of ED and ID to splice-site recognition in a species. On the other hand, there is no clear experimental evidence that suggests how the splice sites of a stretch of CSEs in a nascent transcript are recognized. Intuitively, a long stretch of CSEs, together with its two flanking introns, can be conceived as a whole to be one long intron and the two new splice sites are likely to be separately recognized during two ED-based events. So, counting a stretch of CSEs to be the same as one single-exon skipping event appears to be more reasonable when estimating the CE fraction. Using this modified approach, *G. lucidum*'s CE fraction becomes ordinary when compared to other fungi. The new estimate of CE fraction of *G. lucidum* is lower and is now 0.061 (CE = 16 and RI = 246), which is much closer to that of CE fractions of other fungi; in addition, it only shows slight deviation from the expectation calculated using McGuire *et al.*'s linear model [4].

In addition to the alternative approach described above, excluding CSEs is yet another choice if we believe that CSEs recognition involves a complex combination of splice-site recognition events. For example, it has been shown that, when two antisense oligonucleotides (AONs) are simultaneously use to target two distinct exons, where each AON could induce the skipping of a single exon, a stretch of consecutive seven exons is skipped during transcript splicing of the human Duchenne Muscular Dystrophy gene [55]. This result suggests that the mechanism controlling the splicing of a stretch of CSEs is more complex than expected, implying that it is inappropriate to treat a stretch of CSEs just like a single-exon skipping event. If this factor is included, *G. lucidum*'s CE is further lowered to 0.057. Furthermore, one piece of circumstantial evidence supports the idea that CSEs are different from single-exon CEs in *G. lucidum*. The average length of single-exon CEs is 83 bases, which is close to the average length found in other fungi (17–90 bases). By way of contrast, the average length of the 11 CSEs is about 158 bases. This result is consistent with the argument that CSEs should not be included when estimating CE fractions. All in all, using the two modified estimations for the CE fractions, we conclude that the relative importance of ID and ED in the splice-site recognition in *G. lucidum* is not really different from that of other fungi.

In addition to the one case of CSEs that has just been discussed, there are six other transcript isoforms revealing CSEs that are supported by good EST-genome alignments. However, these six additional cases have been excluded when estimating *G. lucidum*'s CE fraction since each of them is supported by EST-genome alignments containing at least one non-canonical splice site. By following the criteria used previously, the count of CSEs has not been used to estimate the CE fraction. However, it would be interesting to know how such CSEs are regulated in *G. lucidum*, which is a topic that has not been explicitly investigated in fungi.

### Assessment of so-called fruiting-body genes

Since this project has focused on the *G. lucidum* mycelium, the ESTs derived from this study may be able to serve as a negative control for assessing the importance of genes previously reported to be critical to the formation of fruiting bodies. Two outcomes are possible if fruiting body formation involves transcription level control of these genes. First, if such genes are fruiting-body specific, then no corresponding ESTs should have been detected in the *G. lucidum* mycelium. Second, if up-regulation of these genes is critical to the initiation of differentiation from mycelium into fruiting body, there could be a surge in expression of these mRNAs towards the end of the growth period, which would result in significantly more ESTs corresponding to these genes being detected at the later points in the growth cycle.

To assess these possibilities, we searched for previously reported fruiting-body specific expressed sequences and then checked if these sequences were present among the ESTs detected by this mycelium-based project. The *G. lucidum* fruiting-body ESTs (FB ESTs) GO447641 (similar to yeast *Pho84*), GO447972 (similar to yeast *Mob2*), GO447955 (similar to yeast *Profilin*) and GO447282 (similar to yeast *Profilin*) [5] were downloaded from dbEST. The ESTTranscripts with the highest protein-level similarities to yeast *Pho84*, *Mob2*, and *Profilin* are YMGLESTT 52527, 54260, and 53503, respectively (exported from http://ganoderma.ym.edu.tw). Using BLASTN to align each FB EST to the ESTTranscripts, we found that all alignments had high coverage (aligned with >69% of each FB EST length) and very good sequence identity (∼92%). This result suggests that all three fruiting-body genes reported in [5] are also expressed in mycelium. In addition, the counts of corresponding ESTs cloned in this project do not show any obvious excess at any mycelium growth stage or even the end (the 30^th^ day) of the mycelium culture. One possibility that cannot be assessed by this study is whether these so-called fruiting-body specific genes are strongly up-regulated after the 30^th^ day of culture; it is possible that our experimental design did not reach the time point critical to fruiting-body differentiation. To further explore this point, we examined the public-domain *G. lucidum* ESTs in order to find clues. We searched dbEST and found only ∼1000 *G. lucidum* ESTs that were derived from fruiting bodies, as reported by [5]. Since the number was small compared to this project, and no other sets of fruiting body ESTs or expression data are available, it is impossible to determine statistically if these genes are differentially expressed between the mycelium and the fruiting body. Taking the above evidence together, there is no clear evidence from this project to support the hypothesis the genes previously alleged to be fruiting-body specific are true positives.

### Strain compatibility of other G. lucidum ESTs and determining stage-specific genes

We noticed that the fruiting-body ESTs (FB ESTs) downloaded from dbEST showed certain differences at the sequence level to those of the *G. lucidum* genomic sequences as well as to the sequences of the ESTs generated in this project. As many as ∼30% of the “FB ESTs *versus* the reference genome” alignments had identities less than 80%. The patterns of mismatches included short and dispersed indels in putative coding regions. There are two possibilities that may explain this situation. The first is that the sequencing quality of these FB ESTs is not as good as that of the present project and the second is that these FB ESTs were derived from *G. lucidum* strains that were closely related to, but not identical to the ones as used in this project (BCRC 36123 and BCRC 37180). This raises concerns about strain compatibility, since no strain information linked to the annotation of available FB ESTs was available [5]. Since *G. lucidum* is a fungus of high economic values in East Asia, a number of distinctive strains have been selected and cultivated by various farmers. In these circumstances, it seems likely that the differential representation discovered may just reflect inter-strain differences and not necessarily stage-specific regulation. To perform a sensible expression profile analysis in order to find stage-specific genes, we suggest that it is a prerequisite to use transcriptome data derived from the same *G. lucidum* strain.

### Comparison with the findings from another two strains of G. lucidum

During the preparation of this manuscript, the genomes of another two strains of *G. lucidum* have been published [32], [33]. Those projects provide invaluable resources for the studies of *G. lucidum* and their results offer a wealth of insights about the biosynthesis pathways in this species. Chen, S. *et al.* sequenced a dikaryotic strain CGMCC5.0026, which belongs to the *G. lucidum* Asian group [33], whereas Liu, D. *et al.* sequenced a strain that was collected from oak at Hengshan, Hunan province, China [32]. In addition, Chen, S. *et al.* sequenced the transcriptome of *G. lucidum*, generating RNA-seq reads and ESTs of aerial mycelia, primordia, and fruiting bodies [33]. It could be interesting to explore the differences among the results of three independent projects. For example, highly conserved coding sequences across strains may suggest that these genes have important species-specific functional roles. Besides, gene gain/loss or highly variable regions might be associated with strain-specific phenotypes.

To facilitate the following inter-strain comparison, the genome assembly of the strain published by Liu, D. *et al.* is referred to as “Xiangnong”; the genomic contigs of the strain used as the reference in this project is referred to as “BCRC37177”; the chromosomal sequences published by Chen, S. *et al.* are referred to as “G.260125-1”. Xiangnong has the lowest number of predicted genes (∼12,000) [32], while BCRC37177 contains ∼14,000 predicted genes and G.260125-1 contains as many as ∼16,000 predicted genes [33]. In addition to the difference in the numbers of predicted genes, there is considerable diversity among their genome sequences as well as among the protein sequences coded inside. The pairwise similarities of these three strains at the genomic sequence level are ∼85%–∼87%. In the 13,995 protein sequences with at least 30 amino acids predicted in BCRC37177, only 44 and 42 of them are found to have identical counterparts in Xiangnong and G.260125-1, respectively. Less than 40% of proteins (39.9% and 24.8% of 13,995 genes) predicted in BCRC37177 have highly similar counterparts (both coverage and identity > = 90%) in G.260125-1 and Xiangnong, respectively. Besides, when using the protein sequences predicted in G.260125-1 to align with genomic sequences, even fewer identical and high-similarity counterparts were found in BCRC37177 and Xiangnong (data not shown).

Since there are considerable differences among these strains at both the genomic sequence and the protein sequence levels, it is curious to know if different strains of *G. lucidum* synthesize the same set of compounds that have medicinal effects and other interesting properties. It would also be interesting to investigate if different strains share similar regulations implicated in the synthesis pathways of these compounds. We notice that many published researches are likely to have been performed using ATCC strains of *G. lucidum*. For examples, a number of papers have used a *G. lucidum* strain, BCRC 36123 (for examples see [6], [56], [57]). The strains employed in this study, including BCRC 37177 (the monokaryotic strain used for the genome sequencing), the dikaryotic strain used to generate the ESTs (BCRC 36123), and BCRC 37180 (the monokaryotic strain used to generate the ESTs) were all derived from ATCC 32471 (for the sources about the ATCC and BCRC strains link to http://www.straininfo.net/ and https://catalog.bcrc.firdi.org.tw/BSAS_cart/controller?event=WELCOME). This suggests that our study is more likely to provide a transcriptome snapshot of a strain that has been widely studied.

Here we briefly discuss a possible case of the inter-strain differences. A more comprehensive comparison of the genomes of different strains of *G. lucidum* will be performed in a future manuscript dedicated to the annotation and analysis of BCRC37177. We notice that both BCRC37177 and Xiangnong strains are predicted to have a complete set of enzymes required for the synthesis of the core structure of *N*-glycans; however, G.260125-1 is likely to give an exception. The latter strain does not have any predicted genes that have high similarities to the protein sequence of processing A-glucosidase I (GCS1, EC number: 3.2.1.106) (for the corresponding ESTGene found in this project see YMGLESTG49067 in File S9). The best BCRC37177-Xiangnong counterparts of all the genes implicated in the synthesis of the core structure of *N*-glycans, including GCS1, present high sequence similarities (>90%) at the protein sequence level, suggesting that these genes play important biological roles in *G. lucidum*. In particular, the ESTs generated in this study provide solid evidence to support that these genes are expressed in the mycelium of *G. lucidum* (for the counts of ESTs supporting these genes see File S9). GCS1 catalyzes the first trimming step of the protein-bound *N*-glycan precursors. In other species, mutations in *GCS1* genes have been found to lead to abnormal phenotypes such as production of abnormal shrunken seeds in *Arabidopsis* [58]. The biological significance of this missing enzyme in G.260125-1 is yet to be determined. We do not know if there is an isozyme of GCS1 that is of a different evolutionary origin coded in G.260125-1, or the function of this enzyme is unnecessary for the formation of the core *N*-glycan structure in particular strains of *G. lucidum*. Besides, we cannot rule out the possibility that the missing of this gene actually corresponds to a gap yet to be closed in the genome assembly of G.260125-1.

### Availability

All the genomic contigs and ESTs have been submitted to DDBJ and they are to be made public as soon as this manuscript is accepted. The analysis results, including the EST-genome alignments supporting the cassette exons, retained introns, the over-represented ESTGenes, candidate differentially expressed genes, and antisense transcripts, are provided in the supplementary files. In addition, to enable users to access the gene expression information compiled from the CGL ESTs, we have set up the Lingzhi EST project web site (http://csb2.ym.edu.tw/est/). We have also created an Ensembl–based [59] companion web site, http://ganoderma.ym.edu.tw, to help users navigate and compare visually the structures of predicted genes with those of the ESTGenes. The Lingzhi EST project web site provides users with three types of transcription information:

Functional annotation of the ESTGenes
EST-derived alternative splicing information
Mapping of ESTGenes to biological pathways

This web site provides an easy-to-use interface for users to perform keyword searches, to browse the ESTGenes having alternative splice forms, to visualize the ESTs within biological pathways, and to explore sense-and-antisense gene pairs. In addition, “comparative search” allows users who are familiar with *S. cerevisiae* (budding yeast), *S. pombe* (fission yeast), or *A. thaliana* to use gene symbols or keywords from these species to find the most similar genes in the *G. lucidum* genome.

The query/browse result are presented in a tabulated format, in which each ESTGene is listed with its annotation and functional category [29], its involved KEGG pathways [31], the respective numbers of ESTs derived from mycelium on different culture days, and a hyperlink to the Ensembl ContigView (see Figure 1 for an example).

## Supporting Information

File S1 **Length distribution of ESTs.**
(TIF)

File S2 **ESTGenes that are not predicted by GeneMark-ES.**
(XLSX)

File S3 **Overrepresented genes.**
(XLSX)

File S4 **Age-specific overrepresented genes.**
(XLSX)

File S5 **Strain-specific overrepresented genes.**
(XLSX)

File S6 **Anti-sense ESTTranscripts.**
(XLSX)

File S7 **EST-genome alignments containing cassette exons.**
(TXT)

File S8 **EST-genome alignments containing retained introns.**
(TXT)

File S9 **Genes implicated in** ***N*** **-glycan synthesis.**
(XLSX)

File S10 **Hydrophobins.**
(XLSX)

File S11 **The alignment of different hydrophobin proteins.**
(TXT)
