# Correction: Generation and Analysis of the Expressed Sequence Tags from the Mycelium of *Ganoderma lucidum*

**DOI:** 10.1371/annotation/d3ee6fb1-5239-4a09-b197-379d22f27163

**Published:** 2013-09-09

**Authors:** Yen-Hua Huang, Hung-Yi Wu, Keh-Ming Wu, Tze-Tze Liu, Ruey-Fen Liou, Shih-Feng Tsai, Ming-Shi Shiao, Low-Tone Ho, Shean-Shong Tzean, Ueng-Cheng Yang

Two grants from the National Science Council were incorrectly omitted. The Funding Statement should read: "This project was supported by VGH92-378 from the Taipei Veterans General Hospital (http://www.vghtpe.gov.tw/) (Taiwan, Republic of China), and also by NSC99-3112-B-010-019, NSC101-2319-B-010-002, and NSC102-2319-B-010-002 from the National Science Council (http://web1.nsc.gov.tw/) (Taiwan, Republic of China). The funders had no role in study design, data collection and analysis, decision to publish, or preparation of the manuscript." 

