# Supplementary figures and images for: Generation and Analysis of the Expressed Sequence Tags from the Mycelium of Ganoderma lucidum
Source: PLoS One. 2013 May 2;8(5):e61127. doi: 10.1371/journal.pone.0061127 (PMC3642047; doi:10.1371/journal.pone.0061127)

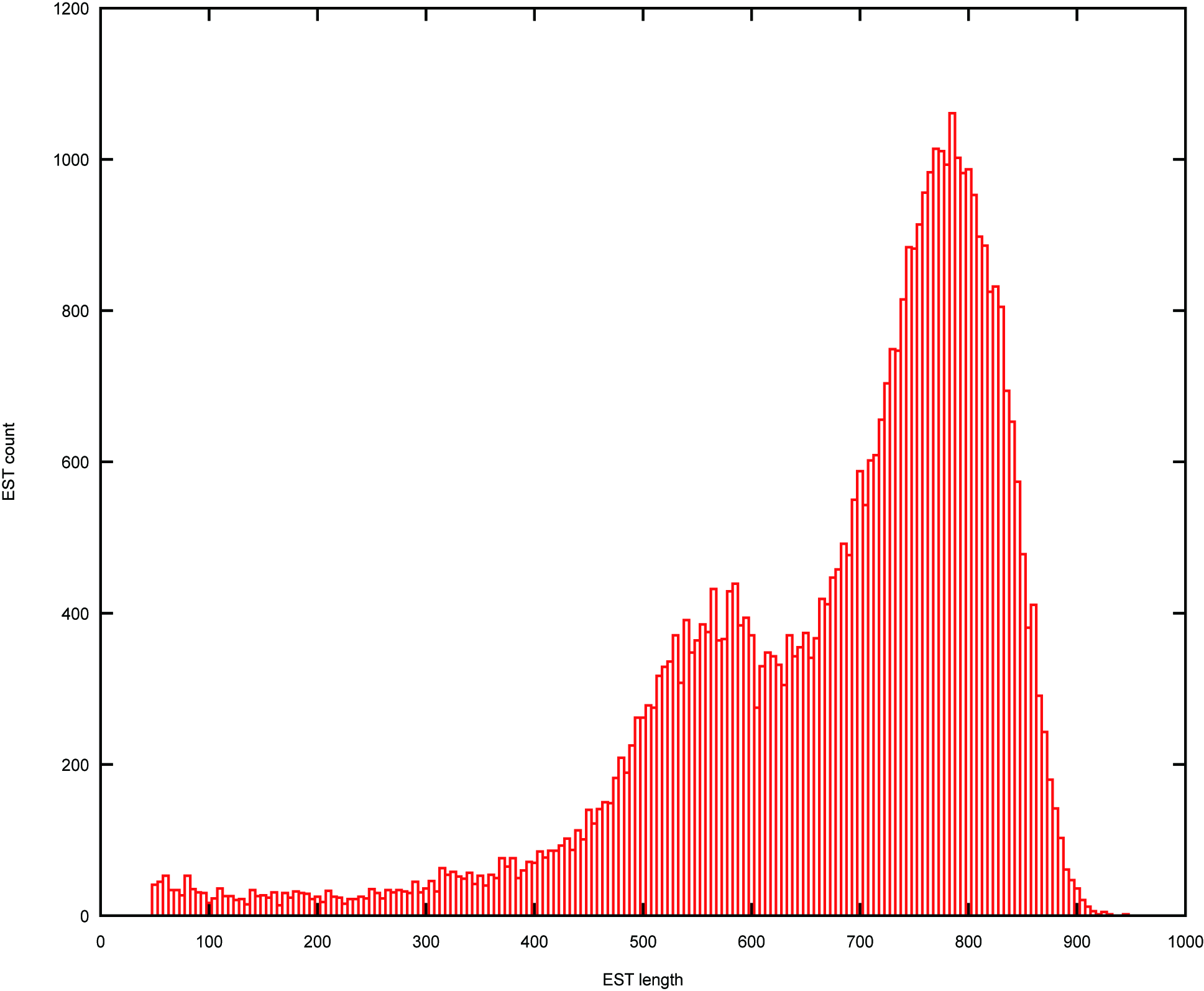

Supplement: File S1 — Length distribution of ESTs. (TIF) [file pone.0061127.s001.tif]
